# Polyanions Cause Protein Destabilization Similar to That in Live Cells

**DOI:** 10.1021/acs.biochem.0c00889

**Published:** 2021-02-26

**Authors:** Therese Sörensen, Sarah Leeb, Jens Danielsson, Mikael Oliveberg

**Affiliations:** Department of Biochemistry and Biophysics, Arrhenius Laboratories of Natural Sciences, Stockholm University, S-106 91 Stockholm, Sweden

## Abstract

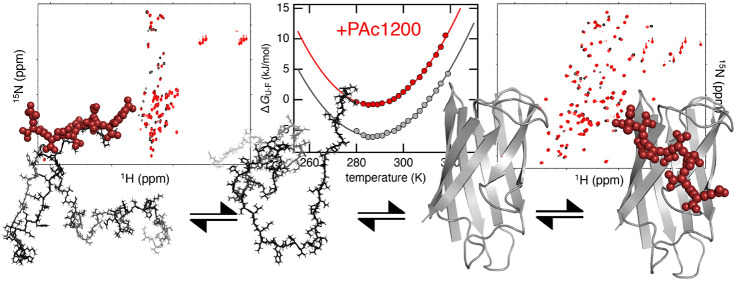

The structural stability of proteins is found to markedly change upon their transfer to the crowded interior of live cells. For some proteins, the stability increases, while for others, it decreases, depending on both the sequence composition and the type of host cell. The mechanism seems to be linked to the strength and conformational bias of the diffusive *in-cell* interactions, where protein charge is found to play a decisive role. Because most proteins, nucleotides, and membranes carry a net-negative charge, the intracellular environment behaves like a polyanionic (*Z*:1) system with electrostatic interactions different from those of standard 1:1 ion solutes. To determine how such polyanion conditions influence protein stability, we use negatively charged polyacetate ions to mimic the net-negatively charged cellular environment. The results show that, per Na^+^ equivalent, polyacetate destabilizes the model protein SOD1^barrel^ significantly more than monoacetate or NaCl. At an equivalent of 100 mM Na^+^, the polyacetate destabilization of SOD1^barrel^ is similar to that observed in live cells. By the combined use of equilibrium thermal denaturation, folding kinetics, and high-resolution nuclear magnetic resonance, this destabilization is primarily assigned to preferential interaction between polyacetate and the globally unfolded protein. This interaction is relatively weak and involves mainly the outermost N-terminal region of unfolded SOD1^barrel^. Our findings point thus to a generic influence of polyanions on protein stability, which adds to the sequence-specific contributions and needs to be considered in the evaluation of *in vivo* data.

The intracellular environment, where the majority of proteins exert their biological function, differs in many respects from the dilute 150 mM NaCl buffer typically used for analysis *in vitro*. Due to diffusive interactions with the surrounding macromolecules,^1−3^ these differences affect not only protein motion but also the structural properties of proteins.^4−7^ One such property is the thermodynamic stability
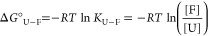
1where [U] and [F] are the concentrations of unfolded and folded protein, respectively,^6,7^*R* is the molar gas constant, and *T* is the absolute temperature.

Previously, the intracellular crowding was assumed to yield a net protein stabilization. This assumption was based on *in vitro* observations showing that excluded-volume effects generally favor compact states.^8^ However, recent studies have shown that the *in-cell* effect varies from protein to protein,^9−12,6^ and that the decisive factors are to be found in the detailed crosstalk between the individual proteins and the intracellular environment.^13,14,3,15^ That is, the change in stability depends on both the protein sequence^3^ and the type of host cells.^14,6^ An illustrative example is provided by the β-barrel of the ALS-associated protein Cu/Zn superoxide dismutase (SOD1^barrel^).^16^ This protein undergoes a distinct stability loss upon being transferred into live human cells (A2780),^6^ which is seen as a decrease in (i) the melting temperature (*T*_m_), (ii) the unfolding enthalpy at *T*_m_ [Δ*H°*_U–F_(*T*_m_)], and (iii) the maximum thermodynamic stability (Figure 1). Overall, this is the thermodynamic signature expected for protein destabilization caused by preferential binding;^17^ i.e., the cellular environment interacts more strongly with the unfolded protein than with the folded protein, shifting the folding equilibrium *K*_U–F_ = [F]/[U] toward U (eq 1). Subsequent transfer of SOD1^barrel^ from human cells to the interior of *Escherichia coli* decreases Δ*H°*_U–F_(*T*_m_) and the maximum stability even further, but now with an accompanying increase in *T*_m_ (Figure 1).^6^ Such a contrasting thermodynamic signature suggests that, on top of the destabilization caused by preferential binding, there is a stabilizing term from excluded-volume effects.^17^ Given that the cytosol of *E. coli* cells is 3–6 times more crowded than that of human cells, this result is perfectly reasonable.^18,5^ The data suggest, interestingly, that the same molecular determinants are at play, both in the native eukaryotic environment and in the *E. coli* cytosol, although in various relative amounts. When it comes to understanding the nature of preferential binding, however, the situation becomes more complicated. Because the average protein, nucleic acid, and membrane carry net-negative charge,^1^ the cellular interior represents basically a polyanionic system with several characteristic features.

**Figure 1:**
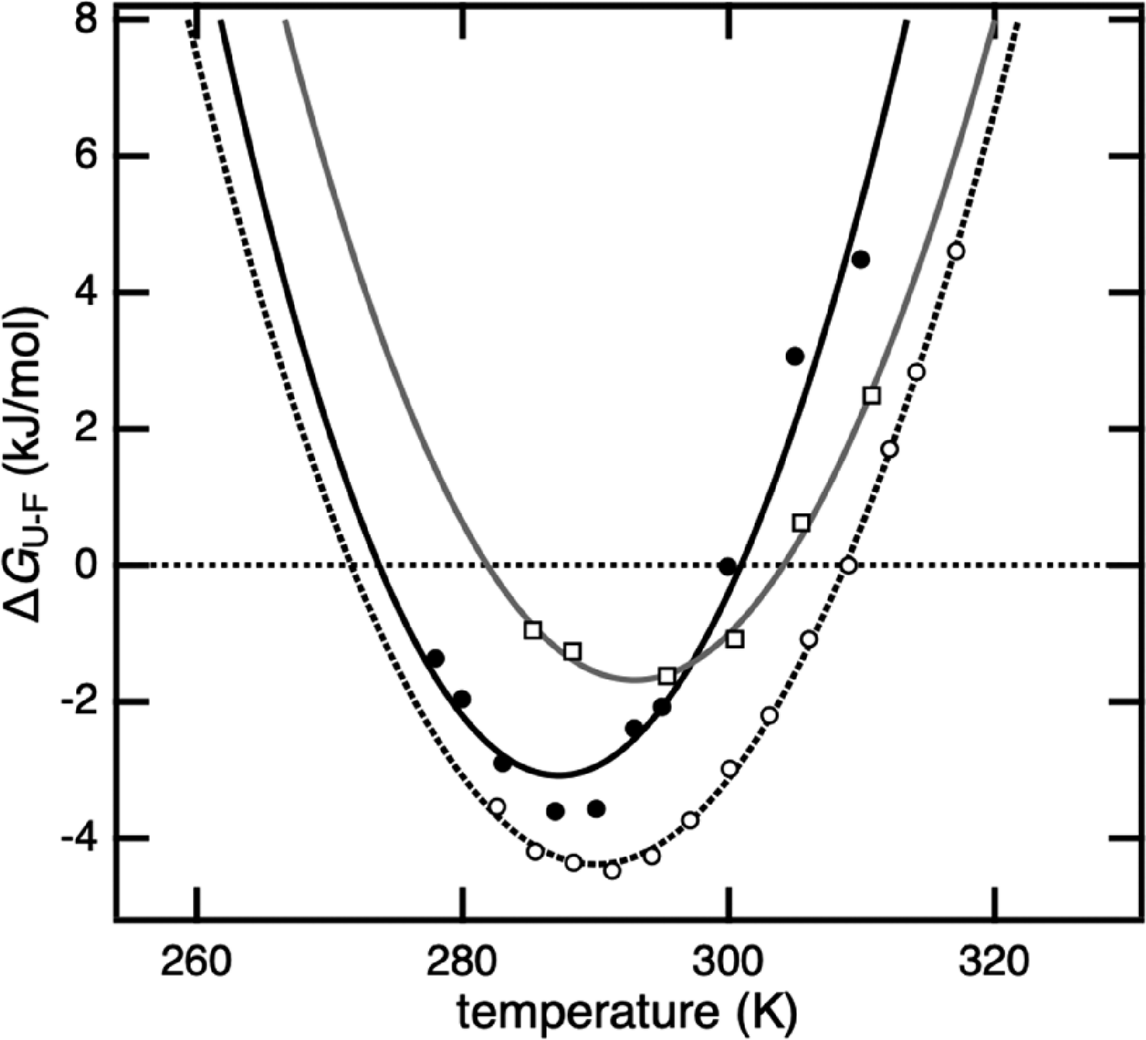
Thermodynamic signature of SOD1^I35A^ in PBS, in the cytosol of human A2780 cells, and in *E. coli* cells. The data are reproduced from ref (6), where a transfer from dilute PBS buffer (dashed line, empty circles) into live human cells (black circles) was associated with a marked destabilization of SOD1^I35A^. Inside the human cells, the protein displayed decreases in both the melting temperature and the maximum stability (Table S1). SOD1^I35A^ was also studied inside *E. coli* cells (empty squares), where the maximum stability decreased even further, as indicated by the upward shift of the thermal stability curve. On the basis of a thermodynamic analysis, it was hypothesized that the observed destabilization inside the two cell types was caused by preferential binding to the unfolded state.^6^ Surprisingly, while the maximum stability was lower, the melting temperature of SOD1^I35A^ increased inside the bacterial cells compared to that inside the human cells. It was further hypothesized that, because the bacterial cytosol is more crowded than its human counterpart, excluded-volume effects contributed with a stabilizing component.

Most notably, the intracellular interactions will experience a global charge repulsion that not only acts at long range but also modulates the close-range binding potential.^1^ Due to the relatively low intracellular levels of small anions, the very reach of the electrostatic interactions will also be longer than in standard 150 mM NaCl buffer.^1^ This repulsive situation will basically oppose association and favor protein dispersion.^1,14^ However, there are also polyion features that are expected to promote binding. For example, elements of covalently linked charges have lower binding entropy than the same number of free charges, which generally favors association.^19^ Likewise, elements with clustered charges are expected to show some degree of binding promiscuity because of their mean field character; i.e., as long as their combined electrostatic fields are attractive, they can associate despite local mismatches.^20^ The frustration of such local mismatches can also add a diffusive component to the associated states and decreased binding entropy cost,^20^ as sometimes observed in biologically optimized binding sites.^21^ In this study, we shed further light on this binding behavior of polyanions by comparing how monoacetate (NaAc) and polyacetate (NaPAc) affect the structural stability of the model protein SOD1^barrel^. The results show that, per negative charge, polyacetate is significantly more destabilizing than monoacetate. At a polyacetate concentration matching 100 mM Na^+^, and at 37 °C, the destabilization is similar to that in mammalian cells. By combining an analysis of the folding kinetics with high-resolution nuclear magnetic resonance (NMR), we further assign the effect to mass action, where the polyacetate interacts more strongly with unfolded SOD1^barrel^ than with folded SOD1^barrel^. This preferential binding is mainly confined to coil segments with positively charged amino acid moieties, which seem to transiently trap the protein in its unfolded state. The findings are discussed in relation to existing *in-cell* NMR data and suggest that the molecular details of transient interactions between a protein and the surrounding molecules must be taken into account when thermodynamic properties are studied inside live cells.

## Materials and Methods

### Mutagenesis, Expression, and Purification of SOD1^barrel^ and SOD1^I35A^

Mutagenesis, expression, and overexpression of SOD1^barrel^ and SOD1^I35A^ have been previously described in refs (16) and (6). In short, genes encoding the proteins were placed in a pET-3a vector and transformed into *E. coli* BL21 DE3 cells. Cells were grown overnight at 37 °C on LB/agar plates with carbenicillin as a selective marker. The obtained colonies were used to seed overexpression cultures. Cultures were grown in LB medium (37 °C) supplemented with 100 μg/mL carbenicillin under shaking until the OD_600_ was 0.8–1.0, after which protein overexpression was induced with 0.5 mM isopropyl β-d-1-thiogalactopyranoside (IPTG). Overexpression proceeded overnight at 23 °C. Upon overexpression of proteins for use in NMR experiments, the culture medium (LB) was replaced with M9 medium with ^15^NH_4_Cl as the sole nitrogen source.

The cells were harvested and lysed using sonication. Cell debris was removed via centrifugation (39000*g*, 20 min, 4 °C) using a JA 25.50 rotor (Beckman Coulter). For SOD1^barrel^, further purification involved heat denaturation at 55 °C, as well as anion exchange and size exclusion chromatography.^16^ The same protocol was used to purify SOD1^I35A^ with the sole exception that, because of the reduced thermostability of SOD1^I35A^, the heat denaturation step was skipped.^6^ Protein purity was verified using 4–20% sodium dodecyl sulfate–polyacrylamide gel electrophoresis gradient gels (Bio-Rad Laboratories Inc.), and the protein concentration was determined using a NanoDrop 2000 (Thermo Scientific). Expression yields were >20 mg/L of cell culture.

### General Experimental Conditions

All experiments were performed at 298 K, with 10 mM MES (pH 6.4) as background buffer, unless otherwise stated. Stock solutions of 2 M NaAc (pH 6.3) (Millipore Sigma) buffer and 2 M NaCl (VWR) solutions were prepared according to standard practices. During preparation of the 2 M polyacetate stock solutions, special consideration had to be taken to reach an ionic strength that was comparable with the salt solutions, as well as a stable pH. The preparation protocol has been extensively described in the Supporting Information.

To avoid excessive screening of charge interactions, as, e.g., observed for GdmCl, we opted here for Ultrapure urea (MP Biomedicals) as the denaturation agent; 5–9 M urea was used for the SOD1^barrel^ unfolding kinetic data, while 5 M urea was used to denature the protein in preparation for the refolding kinetics. When the U state was studied with NMR, 4 M urea and 5 M urea was used as denaturing conditions for SOD1^I35A^ and SOD1^barrel^, respectively. Before use, urea was prepared fresh and had its concentration verified using the refractive index^22^ with a Refracto 30PX portable refractometer (Mettler Toledo).

### NMR Experiments

^1^H–^15^N heteronuclear single-quantum coherence (HSQC) spectra were used for all experiments. Temperature stability data of SOD1^I35A^ were acquired on a Bruker Avance 600 MHz NMR spectrometer, with 2048 increments in the ^1^H dimension, 128 increments in the ^15^N dimension, and 16 scans. The temperature varied between 278 and 318 K in steps of 2 K, with a 10 min equilibration time between each experiment to ensure temperature stability. Data were processed using TopSpin (Bruker).

### Thermal Stability Analysis

The folded (F) and unfolded (U) populations were determined from C-terminal Q110 cross-peak volumes at 8.03 and 125.0 ppm and at 7.97 and 125.4 ppm, respectively^6^ (Figure S2). The Gibbs free energy (Δ*G*_U–F_) was calculated according to eq 1. The temperature dependence of Δ*G*_U–F_ was fitted to^23^
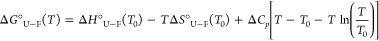
2where *T*_0_ is the reference temperature, Δ*H*°_U–F_(*T*_0_) and Δ*S*°_U–F_(*T*_0_) are the enthalpy and entropy at *T*_0_, respectively, and Δ*C_p_* is the heat capacity difference between U and F. The melting temperature, *T*_m_, and cold unfolding temperature, *T*_c_, were extracted by fitting the thermal stability data to
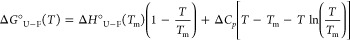
3and obtaining the values where Δ*G*_U–F_ = 0 kJ/mol. Data were analyzed using KaleidaGraph (Synergy Software).

### Analysis of Chemical Shift Perturbations

The chemical shift perturbations (CSPs) of U and F, for SOD^barrel^ and SOD^I35A^, were analyzed under different ionic conditions with and without urea. HSQC experiments were performed on a Bruker Avance 700 MHz spectrometer equipped with a triple-resonance cryoprobe, with 2048 increments in the ^1^H dimension, 128 increments in the ^15^N dimension, and eight scans. Sodium trimethylsilylpropanesulfonate (DSS) was used as a standard reference,^24^ unless otherwise stated. Data were analyzed with Sparky.^25^

CSPs in the ^1^H and ^15^N dimensions were calculated using either 100 mM NaCl (Figure S3) or 100 mM NaAc (Figure 5) as a reference. To show that the two salts are comparable, the CSPs between 100 mM NaAc and 100 mM NaCl were also quantified (Figure S3). The CSPs for ^1^H and ^15^N were normalized according to eq 4,^26^ and the CSPs with normalized values equal to or exceeding two standard deviations (0.02 and 0.04 ppm for U and N, respectively) were considered significant.
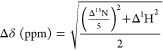
4

### Polyanion Titration Curve, with a Constant Na^+^ Concentration

Data acquisition and chemical shift analysis were carried out as described above. Samples with folded and unfolded SOD1^barrel^ were prepared with 0 μM, 10 μM, 100 μM, 1 mM, 10 mM, 50 mM, and 100 mM NaPAc1200 at pH 6.4. To abolish any unwanted ionic strength effects, the samples were supplemented with NaCl so that the final Na^+^ concentration was 100 mM. Data were analyzed with Sparky.^25^ The apparent dissociation constant (*K*_D_) was determined from the induced chemical shift of the most affected cross-peaks (Figure 5). The induced chemical shifts were fitted assuming a single binding site and *K*_D_. Line widths were quantified by taking the ratio of the peak intensity at *x* mM NaPAc1200 and 0 mM NaPAc1200 (Figure S4).

### Molecular Docking of PAc1200 to SOD1^barrel^

Avogadro^27^ was used to build a visual representation of the 13-mer PAc1200, which was subsequently exported as a Protein Data Bank (PDB) file and further prepared in PyMOL (Schrödinger) to meet the format requirements of the HADDOCK 2.4 Web server.^28^

To dock the PAc1200 molecule to SOD1^barrel^ (PDB entry 4BCZ), both PDB files were uploaded to the HADDOCK Web server via the web interface. Residues identified via NMR CSPs (Figure 5 and Table S2) were treated as active in the interaction. All atoms in the ligand were treated as active. Default settings were used, except for the parameters defined in Table S2. Docking was done without any Na^+^ ions present.

Clusters 1, 5, 8, and 10 received *Z* scores^28^ of −1.9, −0.4, −1.2, and −0.4, respectively, where a more negative *Z* score indicates a better fit. The docking results agree with the diffusive binding hypothesis presented in the text, as illustrated in Figure 6.

### Stopped-Flow Folding Kinetics

Protein folding kinetics were studied using PI-Star 180 and SX.18-MV stopped-flow spectrophotometers (Applied Photophysics), with excitation at 280 nm and emission collected with a 320 nm short-pass filter. Measurements were performed on SOD1^barrel^ in 100 mM NaCl, 100 mM NaAc, 100 mM NaPAc1200, or 100 mM NaPAc8000 at pH 6.4, with 4 μM protein after mixing. The observed folding kinetics were fitted to
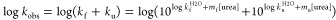
5where *k*_obs_ is the observed rate constant, *k*_f_ and *k*_u_ are the refolding and unfolding rate constants, respectively, log *k*_f_^H2O^ and log *k*_u_^H2O^ are the folding and unfolding rate constants, respectively, extrapolated to 0 M urea, while *m*_f_ and *m*_u_ are the linear urea dependencies of the respective rate constant, describing the change in solvent exposure going from U to the transition state (‡) and from N to ‡, respectively.^29^ Data were analyzed with KaleidaGraph (Synergy Software).

### Mapping Out the Concentration Dependence of (Poly-)ions on the Refolding Rate

Sample preparation and measurements for the concentration curves were as for the stopped-flow refolding kinetic experiments above. The refolding rate of SOD1^barrel^ was measured at NaAc, NaPAc1200, and NaPAc8000 concentrations of 0–1.6 M (Figure S5). The linear increase in refolding rate observed for salt concentrations above approximately 0.35 M (Figure S5) was subtracted to isolate the Debye and binding effects from the general Hoffmeister effects.

### Ionic Strength Control; S6^–17^ Equilibrium Curves

Mutagenesis of wild-type S6 into the supercharged variant S6^–17^ (Figure S1) and the purification protocol have been previously described.^30^ Samples were prepared with 10 mM MES (pH 6.4) as background buffer, 1 μM protein, and 0–5 M urea as the denaturation agent. Samples were allowed to equilibrate overnight before every series of measurements. Measurements were taken on a Cary Eclipse fluorescence spectrophotometer (Varian) with an excitation wavelength of 280 nm. Data were collected by scanning the emission wavelengths of 300–440 nm. Data were quantified via integration over the entire signal, normalized, and plotted as a function of urea concentration.^31^

### Mean Electric Field Distribution

The mean electric field in the unfolded state was calculated assuming that at every position *i*, the total field is the sum of the fields from each residue in the protein chain. The field strength contribution at position *i* from a charge [*q* (1.602 × 10^–19^ C)] at position *j* is then given by
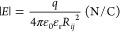
6where *R*_*ij*_ is the distance between residues *i* and *j*, ε_0_ (8.75 × 10^–12^ F m^–1^) is the permittivity in vacuum, and ε_r_ is the relative permittivity, here set to 1. For a flexible unfolded chain, this distance is a distribution of distances. For the simplest model of an unfolded chain, i.e., a Gaussian chain with each amino acid as a chain element, the distance distribution between residues *i* and *j* is given by
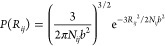
7where *N_ij_* = |*i* – *j*] and *b* is the distance between two adjacent amino acids, here set to 3.88 Å. Combining eqs 8 and 9, integrating over all conformations, and summing over all amino acids yield the total average field at position *i*:
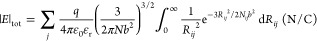
8

## Results

### Outline of the Model System

**Figure 2:**
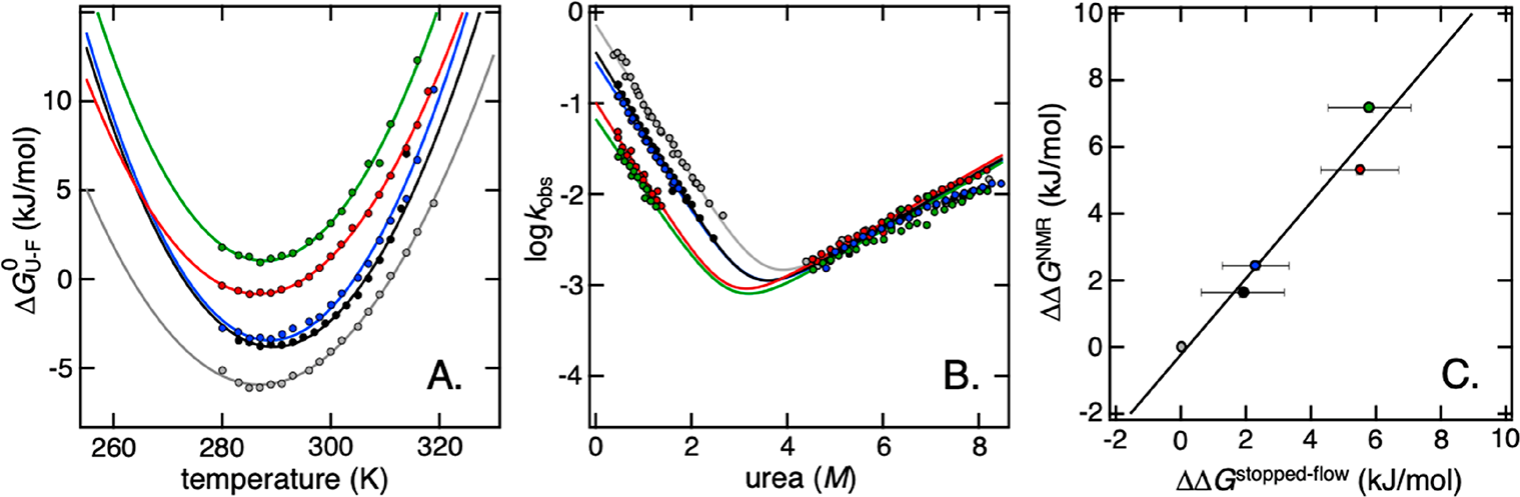
Thermodynamic and kinetic characterization of protein stability under various co-solute conditions. (A) The parabolic curves show the thermal stability of SOD1^I35A^ in different solutes, as detected by NMR. This quantitative approach utilizes the change in U and F populations as a function of temperature to quantify the stability of SOD1^I35A^. It can also be used for subsequent determination of various thermodynamic parameters, and their variation with temperature, according to eqs 2 and 3. The monovalent 1:1 ions NaCl (blue) and acetate (NaAc, black) show a similar, moderate effect, while linking acetate to 13-mers (NaPAc1200, red) and 85-mers (NaPAc8000, green) results in a more severe destabilization. This destabilization is characterized by an upward shift of the curves, revealing a decrease in *T*_m_, a decrease in maximum stability, and an increase in *T*_c_ (Table S1). For comparative purposes, the thermal stability in pure MES buffer (gray) is also shown. (B) Another approach is stopped-flow spectroscopy, where protein folding kinetics are used to quantify the stability of a protein. An analysis of the resulting chevron plots of SOD1^barrel^ reveals a downshift of the refolding limb, meaning that the observed destabilization can be assigned to a reduction in the refolding rate of the protein. (C) Correlation plot of the data shown in panels A and B. Upon comparison of the change in protein stability in the different co-solutes relative to MES (at 25 °C), it can be shown that there is good agreement between the results obtained with the two different methods. All color codes are as in panel A. Error bars reflect the error from data fits in panels A and B.

The focus of this study is to examine how polyanions modulate protein stability by asking the simplest question conceivable: what is the effect of linking a given number of solute 1:1 ions into *Z*:1 ions? As a model protein, we chose the well-characterized SOD1^barrel^ (Figure S1)^3,6,16,32−34^ with the destabilizing core mutation I35A (SOD1^I35A^).^6^ Previous *in-cell* thermodynamic studies of SOD1^barrel^ and SOD1^I35A^ have shown a similar reversible destabilization for these two proteins.^6,35^ The purpose of the I35A mutation is simply to move the thermal unfolding transition into the physiological regime, which allows for a direct characterization of the curved Δ*G°*_U–F_ versus *T* profile (Figure 2), as well as a direct comparison with previously published *in-cell* NMR data.^6^ As ions, we use acetate in the form of “monomeric” NaAc (*M*_w_ = 82.0 g/mol, or 82 Da), the 13-mer NaPAc1200 (*M*_w_ = 1200 Da), and the 85-mer NaPAc8000 (*M*_w_ = 8000 Da), with NaCl as the simplest 1:1 ion reference. Here, the acetate ion is the monomeric counterpart in terms of the charged side chain of polyanionic NaPAc. However, the “true” monomer, as viewed by polymer chemists, would be sodium acrylate (NaAcr). As it turned out, equilibrium stability as well as kinetic measurements proved to be difficult using NaAcr as a reference, because melting experiments with NMR resulted in spontaneous polymerization and a partly irreversible unfolding reaction. The properties of NaAcr also rendered the fluorescence of U and F indistinguishable, effectively obscuring the protein folding and unfolding events. Even so, the existence of a NaAcr effect similar to that of NaPAc can be verified by NMR, showing fully folded and unfolded SOD1^barrel^ in 0 and 5 M urea, respectively. We opted therefore for the acetate ion to represent the monomeric counterpart of the charged NaPAc polymer moieties.

Henceforth, all ion concentrations will be normalized to the Na^+^ concentration to single out the polyion effect, i.e., the total number of charges is kept constant, and the varying factor is *n*, where *n* is the number of linked acetate groups (Figure S1).

### Effects of Polyanions on the Stability of SOD1^barrel^

To obtain a complete thermodynamic fingerprint of the polyanion effect, we mapped out the temperature dependence of SOD1^I35A^ stability by NMR spectroscopy. A simplifying factor is here that the SOD1^barrel^ unfolding transition is a highly concerted two-state process,^16^ with slow exchange between the unfolded and folded states on the NMR time scale.^16^ This enables direct quantification of the equilibrium populations of U and F (*p*_U_ and *p*_F_, respectively) from the NMR cross-peak volumes (Figure S2). For detection, we used the C-terminal residue Q110, which has U and F cross-peaks that are well-separated and exhibits relaxation properties that allow simultaneous determination of *p*_U_ and *p*_F_(6) (Figure S2). From *p*_U_ and *p*_F_, the protein stability (Δ*G°*_U–F_) was calculated according to eq 1 with *K*_U–F_ = [F]/[U] = *p*_F_/*p*_U_. The results show that the temperature dependence of Δ*G°*_U–F_ follows the archetypical parabolic shape in pure 10 mM MES buffer (Figure 2), which is consistent with previous studies of SOD1^I35A^.^6^ Upon addition of 1:1 ions in the form of 100 mM NaCl or NaAc, SOD1^I35A^ undergoes a clear and characteristic destabilization (Figure 2 and Table S1).

**Figure 3:**
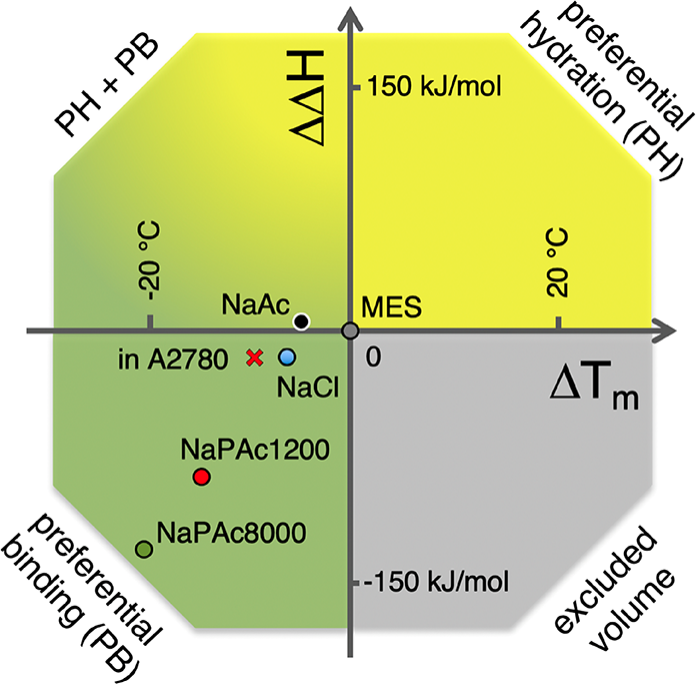
Thermodynamic classification of the destabilization effects from salt and polyanion solutes. Protein destabilization can be classified from the effects on unfolding enthalpy (Δ*H°*) and melting temperature (*T*_m_), following the protocol by Ebbinghaus et al.^36^ Using thermodynamic parameters derived from NMR data (Figure 2 and Table S1), it is possible to determine the major destabilizing factors that act on a protein, i.e., preferential hydration (PH), excluded-volume effects, preferential binding (PB), or a combination of hydration and binding. The 1:1 salts NaCl (blue) and NaAc (black) show small destabilizing effects on SOD1^I35A^, indicating both preferential binding and hydration contributions, while the polyanions (red and green) show distinct preferential binding signatures. For comparison, the effect on SOD1^I35A^ stability in human A2780 cells^14^ is shown as a red x. The figure design was adapted from ref (36).

The difference between the two salts is yet small, with a similar decrease in the maximum *p*_F_ from ∼0.90 in buffer to ∼0.80. This decrease is accompanied by a decrease in *T*_m_ from 38 °C in pure buffer to 31 and 33 °C for NaCl and NaAc, respectively (Table S1). Upon addition of a 100 mM equivalent of the polyanions, however, SOD1^I35A^ destabilization becomes more pronounced. The 13-mer NaPAc1200 yields a maximum *p*_F_ of ∼0.60, with a *T*_m_ of 23 °C, and the 85-mer NaPAc8000 decreases the maximum *p*_F_ to ∼0.43, meaning that the transition midpoint is never reached (Figure 2). Covalent linkage of the 1:1 acetate ions to a 85:1 polyanion thus decreases the maximum SOD1^I35A^ stability from −3.97 kJ/mol (Δ*G*_17.4 °C_^max^) to 1.03 kJ/mol (Δ*G*_14.6 °C_^max^) (Figure 2 and Table S1). Notably, this decrease in maximum stability exceeds that observed in live mammalian cells by a factor of 5.6 (ΔΔ*G*_NaAc-NaPAc8000_^max^ = 5.00 kJ/mol, and ΔΔ*G*_NaAc-A2780_^max^ = 0.89 kJ/mol).^6^ The corresponding effect from NaPAc1200 is a factor of 3.5. Due to a stronger curvature of the free energy profile inside cells (Figure 1),^6^ the destabilization of SOD1^I35A^ by the cytosol and by NaPAc1200 is similar at 37 °C (Table S1). To shed mechanistic light on this polyanion effect, we followed the analysis of Ebbinghaus and co-workers.^17^ In short, in this analysis, parameters Δ*T*_m_ and ΔΔ*H°*_U–F_ can be interpreted as a vector, where the first quadrant (Δ*T*_m_ and ΔΔ*H°*_U–F_ > 0) corresponds to preferential hydration, the second (Δ*T*_m_ < 0, ΔΔ*H°*_U–F_ > 0) to a combination of preferential binding and hydration, the third (Δ*T*_m_ < 0, ΔΔ*H°*_U–F_ < 0) to preferential binding, and the fourth (Δ*T*_m_ > 0, ΔΔ*H°*_U–F_ < 0) to excluded-volume effects (Figure 3).

The derived thermodynamic parameters for the polyanions are both located in the third quadrant (Figure 3 and Table S1). This combination, with negative values for both Δ*T*_m_ and ΔΔ*H°*_U–F_, corresponds to a destabilization due to preferential binding,^17^ meaning that the unfolded state, U, is stabilized by preferential binding to the co-solute. A reason for concern is nonetheless that the 1:1 ions, which are not expected to bind the protein, leave a similar thermodynamic fingerprint (Figures 2 and 3). The likely explanation is that the basal destabilization of SOD1^I35A^, due to ion screening of surface-charge interactions, cannot thermodynamically be distinguished from that of preferential binding, and that the difference lies mainly in the magnitude of the effect.

### Clues from Folding Kinetics: A Selective Effect on the Refolding Limb

The interaction between the ions and the protein species along the folding free energy profile was next determined by stopped-flow kinetics. These experiments were performed with SOD1^barrel^, where the return of an isoleucine at position 35 adds a few methylene moieties inside the protein core, which in turn stabilizes the protein without significantly affecting the charge-based interactions of interest in this study. This extra stabilization allows for a more accurate determination of the refolding kinetics, compared to that of SOD1^I35A^. As typical for proteins that fold via a two-state mechanism,^37^ SOD1^barrel^ displays a v-shaped chevron plot (Figure 2) where the ratio of the unfolding and refolding rate constants (*k*_u_ and *k*_f_, respectively) yields the equilibrium constant (*K*_U–F_) according to
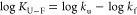
9following the reaction scheme
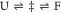
10where ‡ is the folding transition state.

Consistently, the ion-induced stability losses determined from the chevron plots match the NMR data well (Figure 2 and Table S1). A striking feature is yet that the impact is mainly on the refolding rate constants, while the unfolding rate constants are less affected: NaCl and NaAc yield a similar decrease, while the polyanions enhance the effect (Figure 2). The result adds nuance to the picture by showing that the effect of the ion on U is distinct from that of ‡ and F, but it still does not serve to distinguish the preferential binding terms from those of generic charge screening.

### Deconvolution of Ion Binding and Charge Screening

To isolate the generic charge screening from other destabilizing factors, we made use of the characteristic dependence of ion concentration on Debye length.^38^ Commonly, such analysis involves conversion to ionic strength (*I*) and shows typically a saturation above *I* = 500 mM, where the screening effect levels out.^38^ However, because the ionic strength is not easily defined for large polyions, we employ here the equivalent Na^+^ concentrations. As shown below, this simplification turns out to be sufficient for the conclusions drawn.

**Figure 4:**
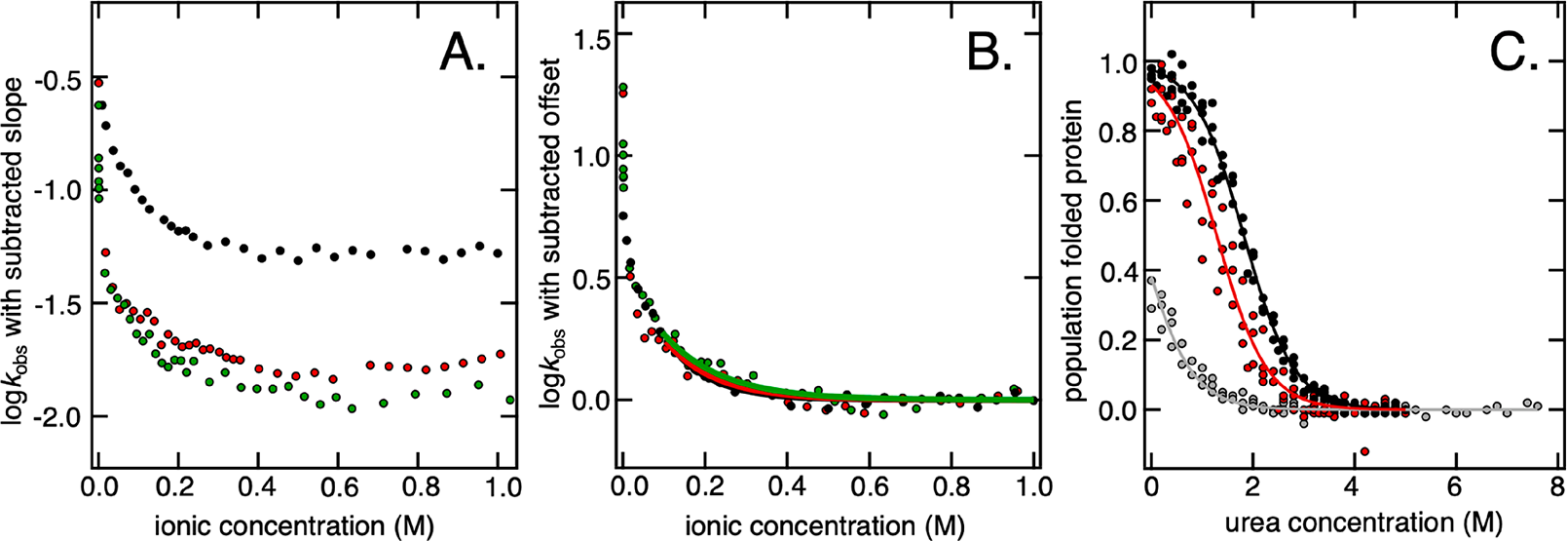
Effects of Debye screening on the refolding rate of SOD1^barrel^. (A) The refolding rate (log *k*_obs_) of SOD1^barrel^ was studied in varying Na^+^ equivalent concentrations of NaAc (black), NaPAc1200 (red), and NaPAc8000 (green). In the data shown here, the Hoffmeister-induced linear increase in log *k*_obs_ at ion concentrations above 0.35 M has been excluded (see Figure S5 for the complete data sets and analysis). In general, the refolding rate of SOD1^barrel^ decreases with an increase in ion concentration, up to approximately 0.4 M, indicating Debye-type screening of charge–charge interactions. On top of the general effects seen for NaAc, a substantial decrease in stability is observed already at very low concentrations of polyanions (NaPAc1200, red; NaPAc8000, green). (B) Same data as in panel A, but with the offset subtracted. The overlapping curves highlight that there are similar Debye screening effects at play at ion concentrations of >100 mM. By fitting single-exponential decays to the data, we can show that the decay constants are virtually the same for all three salts. (C) S6^–17^^30^ was used as an internal control of the ionic strength of the polyanions compared to monovalent salts. The graph shows urea-induced denaturation curves of S6^–17^ in buffer (MES, gray), a monovalent salt (NaAc, black), and a polyanion (NaPAc1200, red). Because S6^–17^ contains only negatively charged side chains, the internal repulsion of the amino acid chain is very strong. However, when salt is added, this internal repulsion is screened, allowing the protein to fold. As the stabilizing effect of equimolar NaPAc1200 is weaker than that of NaAc, we can conclude that the polyanion does not exhibit a higher degree of charge screening than the 1:1 ions.

SOD1^barrel^ displays a slight drop in the refolding rate constant upon titration by moderate amounts of NaCl, and the same initial drop in *k*_f_ is readily reproduced with NaAc (Figure 4). The cause of this destabilization could be screening of nativelike, long-lived electrostatic interactions in the unfolded state, and the drop then corresponds directly to Debye screening.^39−41^ Upon further addition of NaAc, a slow increase in *k*_f_ follows, which is attributed to a general Hoffmeister stabilization^42^ (Figure 4). Consistent with a Debye screening effect at low salt concentrations, the change between the two salt regimes occurs at ∼300 mM.^38^ At this salt concentration, the Debye length is ∼0.6 nm,^38^ which matches the amino acid separation; i.e., the conditions are expected to screen the majority of the protein’s electrostatic interactions. The same trend is observed with the polyanions, but with the difference that the low-salt decrease is 3 times as large (Figure 4). Also, the polyanion destabilization commences earlier than for NaAc: at 18 mM Na^+^ equivalent, the Δlog *k*_f_ values are −0.73 and −0.82 for NaPAc1200 and NaPAc8000, respectively. This can be compared to a Δlog *k*_f_ of −0.17 for NaAc (Figure 4), and the observed difference is in good accordance with the findings from stopped-flow kinetics (Figure 2). This result is inconsistent with a Debye screening effect alone, as its final magnitude is primarily determined by the protein’s electrostatics and, hence, largely insensitive to this type of solute ion. On this basis, we conclude that a major part of the destabilization caused by the polyanions has a mechanistic origin different from that of charge screening. That is, there are two superimposed factors at play.

To obtain an independent control of the polyanion screening effect, we monitored their influence on the stability of a supercharged variant of the ribosomal protein S6 [S6^–17^ (Figure S1)].^30^ A useful feature of S6^–17^ is that it contains only negatively charged side chains and, hence, suffers a high level of internal destabilization from electrostatic repulsion.^30^ The uniformly negative surface of this protein also suppresses binding with any solute anions, biasing the readout as far as possible to charge screening effects. Our test shows that, despite the different anionic properties of NaAc and NaPAc1200, they stabilize S6^–17^ to similar extents. Upon addition of 100 mM Na^+^ equivalent, the urea-induced unfolding midpoint increases from −0.25 to 1.28 M (Figure 4), which corresponds to a protein stabilization of 7.5 kJ/mol and matches well the effect of 100 mM NaCl reported previously.^30^ Taken together, the similar screening characteristics of the different ions corroborate the idea that the stronger destabilizing impact of the polyanions on the stability of SOD1^barrel^ originates from mass action through preferential binding to the unfolded state.

### Mapping Out the Interactions between the Polyanions and Unfolded and Folded SOD1^barrel^

The structural foci for the interactions between polyanions and globally unfolded SOD1^barrel^ were determined by high-resolution NMR. To ensure full population of the unfolded state, the protein was further destabilized by the addition of 5 M urea. The noncharged urea is not expected to significantly alter the electrostatic interactions in the system, even though it might suppress binding by weakening attractive polar and hydrophobic contacts between the polyanions and the random coil. In such case, the urea will lead to an underestimation of binding compared to the pure situation. Because a similar magnitude of destabilization is observed in the NMR temperature scans and the urea-titrated folding kinetics, however, the mechanism appears to be relatively unaffected by urea.

**Figure 5:**
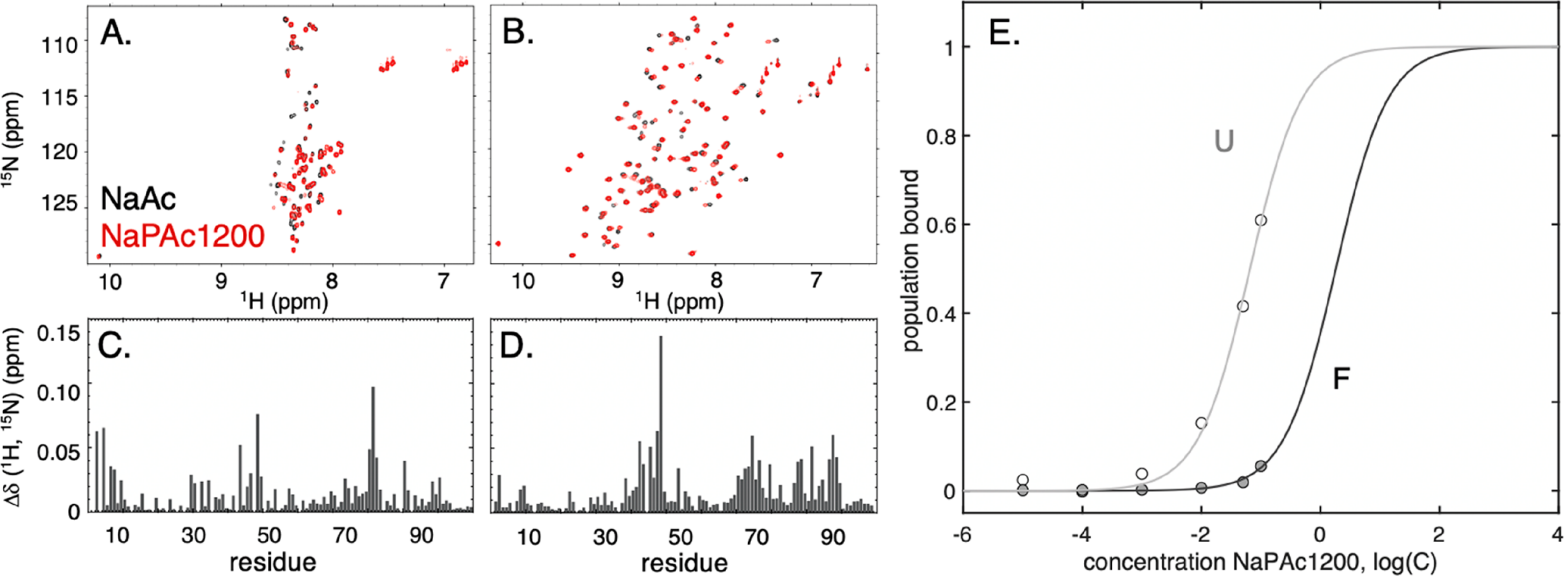
NMR characterization of binding of NaPAc1200 to the folded and unfolded state of SOD1^barrel^. (A) Overlapped NMR spectra of unfolded SOD1^I35A^ (U) in 4 M urea, with (red) and without (black) 100 mM Na^+^ equivalents of NaPAc1200. A comparison of the spectra reveals small but significant shifts. (B) Corresponding NMR spectra of folded SOD1^barrel^ (F). Because line broadening is observed for NaPAc1200, the contours in panels A and B have been optimized for peak visibility and may therefore differ. (C and D) Induced chemical shift perturbations upon interaction with NaPAc1200 for U and F, respectively. The reported shifts are the weighted averages of the induced ^1^H and ^15^N chemical shift perturbations. (E) SOD1^barrel^ was titrated with 0 to 100 mM Na^+^ equivalents of NaPAc1200, at a constant Na^+^ concentration. The titration was performed in the presence (U) and absence (F) of 5 M urea. The affinity of the polyanion for the protein was estimated from fitting a binding curve to the chemical shift data, yielding dissociation constants (*K*_D_) of 65 mM for U and 1.8 M for F.

Urea-unfolded SOD1^barrel^ reveals a characteristic ^1^H–^15^N HSQC spectrum with the backbone amide resonances collapsed into a narrow region, especially in the proton dimension, where all cross-peaks are located in the range of 7.9–8.7 ppm (Figure 5). To set the baseline for unspecific ion effects, we first subjected the protein to 100 mM NaCl. As a result, minute general shifts are found along the whole sequence with somewhat more pronounced effects on segments corresponding to strands 4 and 7 (Figure S3). Replacing NaCl with equivalent amounts of NaAc induced no observable chemical shift changes compared to NaCl (Figure S3). Next, when the 100 mM acetate anions are “linked” into 100 mM Na^+^ equivalent NaPAc1200, nine of 110 residues in the protein undergo significant chemical shift changes.

Interestingly, these changes also involve new parts of the protein sequence, compared to monomeric NaAc (Figure 5). The affected residues can be categorized into two groups. The first group shows the involvement of the first 10 amino acids of the SOD1^barrel^ sequence, where five residues in the N-terminal region show consistent polyanion perturbation (Figure 5). The second group comprises the protein’s five histidine side chains that reside more scattered in sequence at positions 43, 46, 48, 80, and 90 (Figure 5). However, the chemical shifts of histidine residues are sensitive to their protonation states,^43,44^ and because the detailed screening properties of monovalent salts and polyanions are expected to be different, these shifts can simply reflect small shifts in the imidazole protonation states; i.e., they need not report on specific binding events. This leaves a polyanionic interaction targeting preferentially the N-terminal region of the unfolded SOD1^barrel^ sequence. In contrast to the similar direction of the screening and binding effects indicated by thermodynamic analysis (Figures 2 and 3), the NMR chemical shifts display distinct chemical shift signatures induced by 1:1 ions and the polyanions, respectively. This indicates that there are two components to the destabilization, where the second relates to binding, and where binding to the unfolded state causes a destabilization by a population shift toward U (eq 10) by mass action.

**Figure 6:**
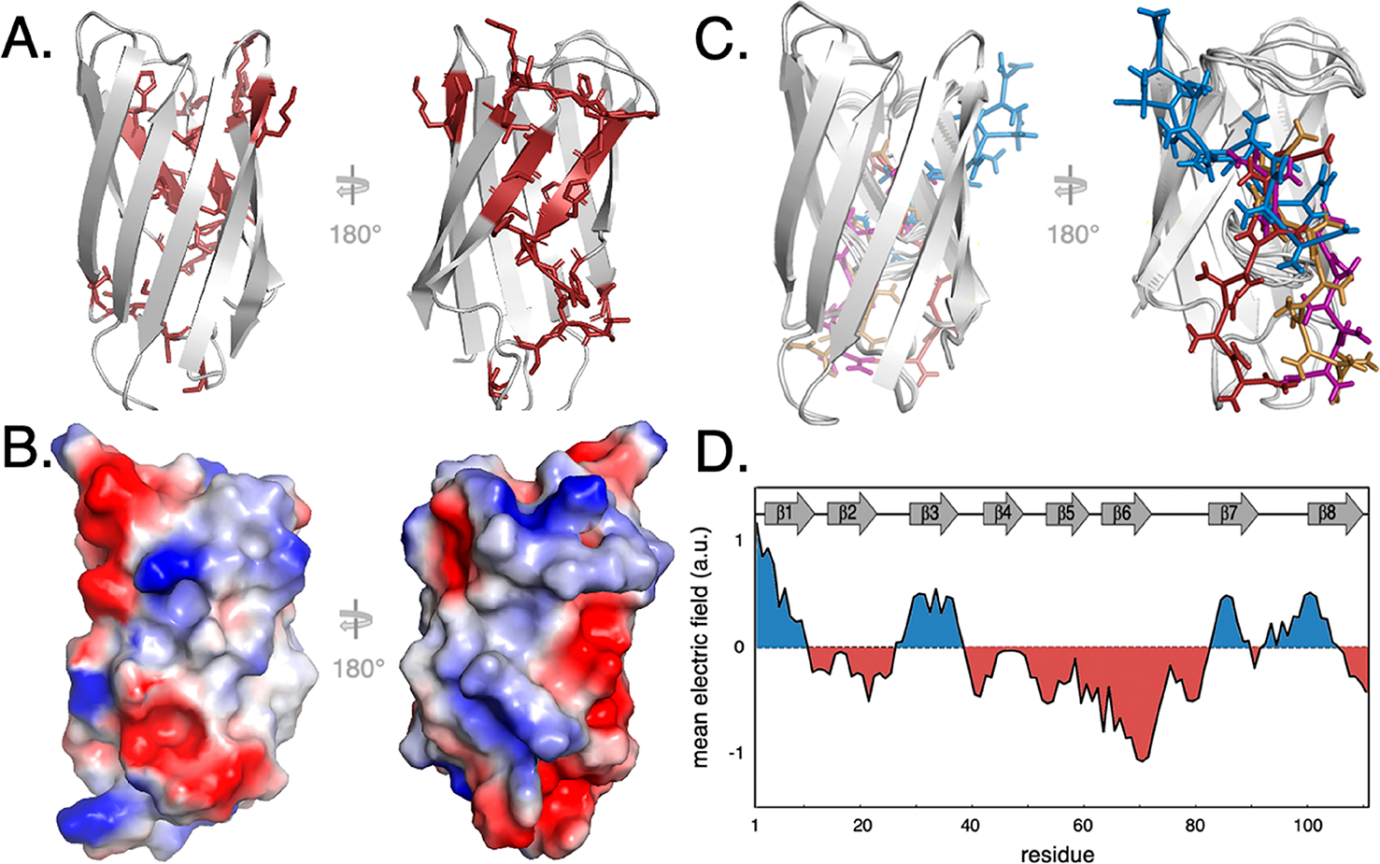
Binding of NaPAc1200 to the folded state. (A) Residues that show significant chemical shift perturbations are colored red in the SOD1^barrel^ tertiary structure, revealing residue co-localization. (B) The electrostatic surface properties of SOD1^barrel^ are projected onto the surface of the protein, indicating that the negatively charged NaPAc1200 binds a groove with mainly positive charges (blue). (C) Five of the best scoring dockings of NaPAc1200 (colored) are shown bound to SOD1^barrel^ (gray), confirming that the binding may be delocalized. (D) Mean field electric field strength calculated for each residue in the unfolded state (eqs 6–9). The mean field pattern was estimated using ordinary Coulombic electrostatics under the assumption that the U state can be approximated as an ergodic Gaussian chain. Positive patches are colored blue, and negative patches red. The positions of secondary structure elements along the protein sequence are shown as a cartoon.

The question is then the extent to which the polyanions interact with the folded state of SOD1^barrel^, and therewith provide a compensatory stabilizing component to the folding equilibrium in eq 10. To find out, we repeated the NMR analysis described above in the absence of urea, assuring full population of folded SOD1^barrel^. The results show that the folded SOD1^barrel^ spectrum also displays significant chemical shift changes in the presence of polyanions. More precisely, the effect is observed in the region between residues 44 and 51. There are also four additional regions of sequence with somewhat less shifted cross-peaks (Figure 5), and notably, all five identified regions join up as a contiguous belt when projected onto the SOD1^barrel^ tertiary structure (Figure 6). This belt spans >3 nm across the protein’s surface, covering β-strands 4 and 7 of the catalytic site, as well as loops 4, 6, and 7.

An interesting detail is here that the span of 3 nm exceeds the length of the fully extended NaPAc1200 13-mer, pointing to the possibility that the binding is to some extent delocalized or involves more than one polyanion molecule. To test the hypothesis of delocalized binding, we employed the chemical shifts as constraints for a docking simulation, using the High Ambiguity Driven protein–protein DOCKing (HADDOCK) server.^28^ We found that several binding modes agree with the constraints, and overlaying five of the best scoring docking results nicely cover the perturbed belt (Figure 6). We also observe that some of the affected cross-peaks exhibit not only induced chemical shift changes but also line width effects (Figure S4). This feature indicates that the polyanion alters some local backbone dynamics in the adjacent loops upon interaction, although exchange contributions cannot be ruled out.^45^ Most affected are the flexible loops, where loop 6 displays larger than average line broadening, while loop 7 displays line sharpening (Figure S4). As both of these regions are dynamic on several time scales in the absence of polyanions,^16,34^ their configurations are inherently sensitive to any perturbation of the protein’s active site region.

This leaves us with a situation in which the polyanions bind both the globally unfolded (U) and fully folded (F) SOD1^barrel^, giving opposing mass-action terms: binding to U causes destabilization, whereas binding to F is stabilizing. Determination of the net effect requires thus information about the relative affinities, i.e., which state binds the polyanion more strongly.

### Polyacetate Shows the Highest Affinity for the Unfolded Protein

From the law of mass action, protein destabilization occurs only when the ligands favor U over F. This can occur through either a higher affinity or a larger number of binding sites. To find out which, we conducted a simple titration experiment in which the polyanion concentration was varied between equivalents of 0 and 100 mM Na^+^. Concentrations of ≫100 mM precluded accurate NMR detection due to the high ionic strength.^46^ Beginning with unfolded SOD1^barrel^, the results show that the five outermost N-terminal residues titrate in parallel, with a chemical shift transition emerging above 1 mM (Figure 5). This concerted response suggests that the N-terminal region of the unfolded chain reports on a single binding event. Even so, it is clear from the lack of a final baseline that the experiment is short of reaching full binding. However, under the assumption that the binding curve is sigmoidal and involves one polyanion (*m* = 1), we can still draw some conclusions about the affinity from estimates of the infliction point (*Mp*_T_), i.e., the transition midpoint where the second derivative passes zero. The relative population change is smaller between 50 and 100 mM than between 10 and 50 mM, indicating that the midpoint has already been passed at 100 mM. Fitting of a sigmoidal function with *m* = 1 yields *K*_D_ ≈ 65 mM Na^+^ equivalent. A corresponding titration of folded SOD1^barrel^ reveals similarly concerted chemical shift changes, but with a binding curve shifted toward higher polyanion concentrations (Figure 5). Inspection of the curve indicates that *Mp*_T_ seems to fall far above 100 mM. This concurs with an affinity that is at least 1 order of magnitude weaker than that for the unfolded protein, i.e., *K*_D_ ≈ 1.8 M (Figure 5). It is clear from the fit that this affinity is encumbered with large uncertainties, further enhanced by omitting the possibilities of both higher-order phenomena and multiple binding sites. Nonetheless, the results are fully consistent with our kinetic and thermodynamic analysis and, hence, do not falsify our minimalist binding model. In other words, on top of the generic charge screening effects, polyanions appear to modulate protein stability by preferential binding.

## Discussion

### Folding Causes the Polyanion Binding to Swap Sites

Just like proteins remain functionally dispersed in crowded cells, the association between our polyanions (−13 to −85 *e*) and SOD1^barrel^ (−0.7 *e*) is basically unfavored by net-charge repulsion. Even so, this net-repulsive term can be overcome at close range, where the local side chain contributions tend to dominate the binding potential.^47^ Starting with the folded SOD1^barrel^ structure, the chemical shifts induced by the PAc1200 polyanion are found in the positively charged groove defined by loops 6 and 7 (Figure 6). This groove is favored over other positively charged surface positions perhaps because it presents the most contiguous positive field and it provides an entropic advantage in the form of multiple, overlapping binding configurations. In other words, compared to other grooves, it allows for a higher number of alternative binding options, which is supported by molecular docking data (Figure 6).^28^ For comparison, attaching the positively charged cell-penetrating TAT peptide to loop III of SOD1^barrel^ induces a similar chemical shift pattern on the protein surface that runs perpendicular to that of the polyanion.^32^ This pattern corresponds to a more negatively charged groove, thereby enabling binding of the linked TAT peptide.^32^

When it comes to the interaction between the polyanion and unfolded SOD1^barrel^, the electrostatic terms are more complex to pin down. As a substitute for a well-defined structure, we simply calculated here the average electric field along the protein sequence using a Gaussian chain approximation, where the distance between two residues along the chain is normally distributed (eqs 6–8 and Figure 6). The results indicate four sequence regions with positive fields. These putative binding sites for the polyanion are along β1, β3, β7, and β8, while NMR analysis confirms chemical shift perturbations induced by the polyanion mainly in N-terminal strand β1 (Figure 5). The reason for the β1 binding preference is not clear but indicates that factors other than the average electric field are at play, e.g., higher structural flexibility toward the termini, where the N-terminis shows a positive field, while the C-terminus does not. Such high dynamics can possibly allow for higher malleability in polyanion binding.^48^ Naturally, the SOD1^barrel^ coil can also harbor structural propensities missed by the simple Gaussian chain approximation, explaining the observed preference for interaction of the polyion with the disordered N-terminal region. A crucial detail is that the observed binding site in the unfolded protein is different from that in the folded protein; i.e., the polyanion needs to swap from one site to another in the U ⇌ F transition (eq 10).

### Protein Destabilization from Mutually Exclusive Binding Sites

**Figure 7:**
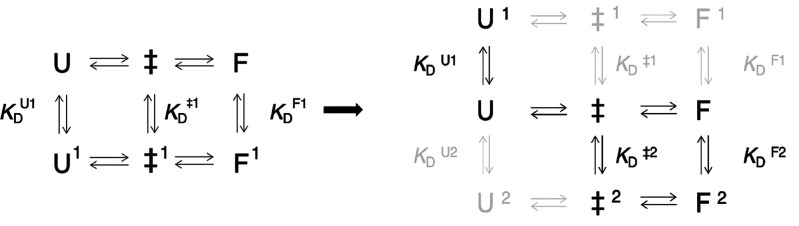
Thermodynamic schemes for polyanion binding. For a system in which the binding site is the same for both U and F, the system contains six states, including the transition states, which can interconvert as shown at the left. In the case of SOD1^barrel^ binding to the NaPAc1200 polyanion, there are two putative binding sites, and the thermodynamic scheme therefore grows to nine states (including the transition states). Here, only one distinct binding site is available for U and F, and direct transitions from F^1^ to U^1^ or F^2^ to U^2^ are forbidden. This reduces the allowed number of states from nine to six, as shown at the right.

In the simplest case, protein destabilization by single-ligand binding is modeled by a coupled six-state equilibrium^49^ (Figure 7). The ligand interaction is here defined by a single binding site with different dissociation constants in the unfolded, folded, and transition state species (*K*_D_^U1^, *K*_D_^F1^, and *K*_D_^‡1^, respectively) (Figure 7). Although this six-state description accurately captures the effects of, e.g., metal coordination and single-side chain protonation,^49−52^ it fails to account for the polyanion data. The reason is that SOD1^barrel^ displays two distinct binding sites, i.e., the unfolded N-terminus and the active site sheet (Figures 5 and 6). This two-site extension yields formally nine interconverting states, including the folding barriers (Figure 7). Because the unfolded β1 and folded surface binding sites are effectively state specific, i.e., site 1 is available only in U and site 2 only in F, the model reduces to six thermodynamically connected species (Figure 7). The reduction also includes ‡^1^, as the kinetic data suggest that the dominant folding pathway is over ‡ (Figure 7). In other words, the polyanion detaches in the unfolded ground state, yielding a selective decrease in the refolding rate constant (Figure 2 and Table S1). For a more detailed mechanistic description and quantification using this minimal binding model, see the Supporting Information.

### Implications for the Intracellular Interactions

To maintain cellular function, proteins display great diversity in structure, dynamic flexibility, and physicochemical properties. Part of this diversity is to ensure specificity for the control of complex biological processes, and these protein features are typically highly conserved across organisms.^1^ The system, however, also needs to avoid unspecific collapse or, at least, keep the competing nonspecific interactions at bay.^53^ Recent findings have shown that this “background” tuning is coded into the sequence regions that are generally considered nonconserved, i.e., in the variable protein surfaces outside of the active sites and specific binding interfaces.^3,14^ Interestingly, these evolutionarily variable parts of proteomes also make up a major part of the exposed intracellular surfaces and dictate thus the system’s “colloidal” stability and diffusive behavior.^1,14^ An important term is here the repulsive net-negative charge,^1,3^ which seems to keep the random protein–protein interactions swift and reversible by balancing out promiscuous attractive forces at short and medium range.^1^ However, there is more to it. From inspection of any nonconserved protein surface, it is clear that it is decorated with a mixture of negative and positive charges (Figure 6), and that these charges naturally interact upon a protein–protein encounter. The charge distribution appears typically quite even, whereas polarizations into contiguous regions of uniform charge are overall rare, unless they are functionally conserved.^14,54,55^ Such mixed and evenly distributed patterns are expected to minimize the chance of attractive match upon collision, simply because of a high likelihood of local conflicts in the charge pairing. Following the idea of gatekeeping,^30^ such surfaces may be the product of rounds of negative-design events; i.e., fitness is increased by point mutations that obstruct a certain interaction interface. Because evolution of new beneficial interactions is nonetheless favored by a system that is maintained at the “brink of collapse”, the existing charge patterns must to some extent be kept promiscuous.^1,53^ That is, the beneficial impact of random mutations needs to be decisive enough to ensure adaptation. It is conceivable that the polyanion binding to the surface of folded SOD1^barrel^ reflects such compromises in the surface-charge distribution, which in this case is a somewhat polarized spot containing mainly positive side chains (Figure 6). Although the present polarization is partly a result of “unnatural” loop truncation,^7^ the result serves to illustrate the importance of surface-charge details in promiscuous binding. Consistently, the positively charged TAT peptide follows the same principle, using a more negative charge polarization next to the PAc1200 binding site.^32^ When it comes to implications for protein–protein interactions *in vivo*, it is clear that densely charged polyanions possess an electric field that is both stronger and more condensed than in the canonical globular protein. The observed polyanion binding can thus shed light on the limits of diffusive charge–charge interactions or even exemplify an indiscriminate trap. Consistently, natural proteins display overall more frustrated electrostatic fields with mixtures of positive charges that prevent such traps in favor of swift diffusion and specific target control.

The question is then how this situation changes upon global unfolding. Even if the structural features of the disordered SOD1^barrel^ remain elusive, it is clear that the very flexibility of the unfolded state will increase the number of binding possibilities. The loss of configurational entropy involved in forming any of these binding sites will naturally lower the affinity, but this penalty may be compensated by better charge pairing, as well as some entropy gain from the flexibility of the interaction itself. As indicated by the data, these compensatory features still promote an affinity that is higher than that for the folded protein. The higher affinity of the unfolded protein for charged polyanions also sheds light on the mechanism of the observed *in-cell* destabilization:^6^ due to its higher flexibility, the coil can find stronger fits to the surrounding molecules than the rigid native state. Although the binding targets in the intracellular milieu are different from the polyanion, they do share some of its features in the form of locally clustered charge distributions and flexible loops. In summary, our observations indicate thus a semispecific type of interaction that seems to rely on charge cluster attraction rather than on precise sequence identity. As the interaction is also strong enough to modulate protein stability, its biological occurrence and possible role in cellular function calls for further elucidation.

## Supporting Information Available

Preparation of polyacetate stock solutions, quantitative test of the binding model in Figure 7, molecular structures of proteins and ionic compounds used in this study, quantification of the NMR temperature scan data, comparison of NMR spectra and chemical shift perturbations in different salt environments, NaPAc1200-induced line width effects in folded and unfolded SOD1^barrel^, control experiments with varying Na^+^ concentrations, thermodynamic parameters derived from NMR temperature scans and folding kinetics, and docking of PAc1200 to SOD1^barrel^ in HADDOCK 2.4 (PDF)

## Accession Codes

The protein in this study is a derivative of the human superoxide dismutase 1 (UniProtKB P00441). This variant has truncated loops where residues 49–91 and 124–139 are replaced by Gly-Ala-Gly linkers. In addition, the C6A, C111S, and C146S point mutations are introduced to avoid disulfide linkage.^16^ In the control experiments, ribosomal protein S6 from *Thermus thermophilus* was used (UniProtKB Q5SLP8). In this variant, all K and R side chains were replaced with serine side chains, resulting in a very negative protein.^30^
