# Supplementary material for: Polyanions Cause Protein Destabilization Similar to That in Live Cells
Source: Biochemistry. 2021 Feb 26;60(10):735–46. doi: 10.1021/acs.biochem.0c00889 (PMC8028048; doi:10.1021/acs.biochem.0c00889)
Supplement: Supplementary file 1 — bi0c00889_si_001.pdf [file bi0c00889_si_001.pdf]

Supporting Information

Polyanions cause protein destabilisation similar to that in live cells

THERESE SÖRENSEN, SARAH LEEB, JENS DANIELSSON* AND MIKAEL OLIVEBERG*

Department of Biochemistry and Biophysics, Arrhenius Laboratories of Natural Sciences, Stockholm University, S-106 91 Stockholm, Sweden.

*Corresponding authors: Jens Danielsson and Mikael Oliveberg

E-mail: mikael@dbb.su.se, jens.danielsson@dbb.su.se

Supporting experimental procedures

Preparation of poly-acetate stock solutions. The polymers NaPAc1200 (product id: 416010, Fig. S1) and NaPAc8000 (product id: 416029, Fig. S1) were purchased from Sigma-Aldrich (Millipore Sigma). In order to produce NaPAc solutions with an ionic strength comparable to that of the monovalent salt solutions (NaCl and NaAc), it was first assumed that all negatively charged subunits in the polymers are associated with a positively charged counterion (Na^+). By dividing the molecular weight of each polymer (1200 g/mol or 8000 g/mol) with the molecular weight of the corresponding monomer (94.05 g/mol) the number of subunits were obtained (~ 13 and ~ 85). Note that, when manufacturing the polyanions the molecular weights of the purchased solutions refer to the average of a distribution of weights of the polyanions inside the bottles. Therefore, the solutions may also contain polymers that are slightly longer or shorter than the calculated lengths (number of subunits). However, this is not expected to have any bearing on the results presented here and it will therefore be disregarded.

The listed densities of the purchased stock solutions and their refractive indexes were used to calculate the stock polymer concentration, and from that the monomer concentration could be further calculated. Using the obtained value, new stock solutions were prepared with 2 M monomer concentration. Because NaPAc1200 and NaPAc8000 have a higher pH (7.4-7.8) than the experimental pH (6.4), and because their buffer capacities are higher than the background buffer used in the experiments (10 mM MES), both polymer stock solutions had their pH adjusted to 6.3 using HCl (VWR). At any subsequent dilution steps the pH would always be verified and corrected as needed using a SevenCompact pH meter (Mettler Toledo). It should also be noted that both proteins used in these experiments, $SOD1^{barrel}$ and $SOD1^{I35A}$, contribute with buffering capacity, and addition of them generally increased the sample pH with 0.1. Therefore, the pH of the samples was verified before every experiment to ensure pH 6.3-6.4. By preparing the polyanion solutions this way, all solutions used in the experiments are expected to have the same amount of charges present, and therefore the same ionic strength. All data presented in the main text have polymer concentrations that refer to the concentration of monomeric subunits present in the solution, or '[Na^+]-equivalents'.

Supporting Information

Quantitative test of the binding model in Figure 7. To put this model to test, we first compared the population of polyanion bound U obtained from NMR with that inferred from the refolding kinetics (Fig. 2 and Fig. 5). Using K_D^{U1} = 65 mM from NMR titration data, the population of bound unfolded species (U^1) is calculated to p_B = 0.61 at 100 mM Na^+ equivalent NaPAc1200. The corresponding number derived from the refolding kinetics is p_B = 0.65. Note that this is from a simple three state model where only the stronger binding to U is taken into account. Considering that K_D is estimated from a titration without final baseline, the agreement between the independent measures is notably good. Next, using the obtained K_D^U and K_D^F to estimate the expected total destabilization in a simple four state mass action model yields $\Delta\Delta G_{U\text{-}F}$ = 2.1 kJ/mol compared to $\Delta\Delta G_{U\text{-}F}$ = 3.5 kJ/mol from kinetic and NMR data, fully consistent with the population difference determined above. In summary, our analysis shows that the observed destabilisation of $SOD1^{barrel}$ by poly-acetate can be accounted for by preferential binding to U, causing a mass-action shift to higher populations of unfolded material.

Supporting Figures

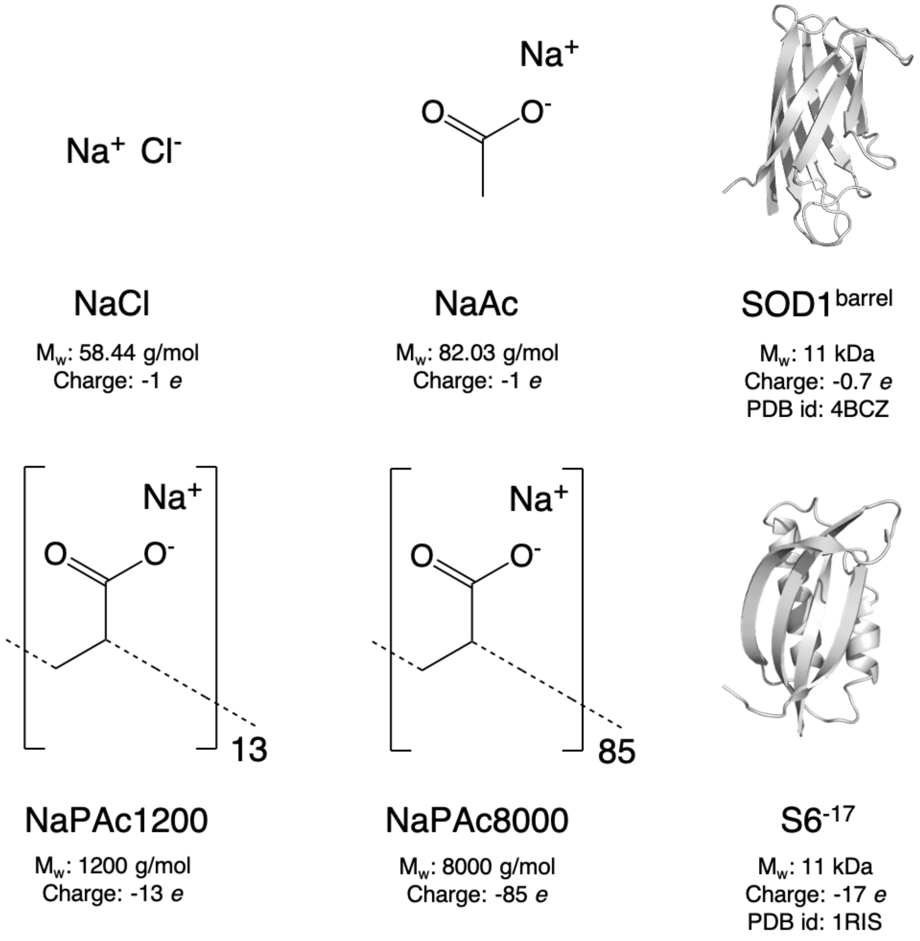

Figure S1. *Molecular structures of proteins and ionic compounds used in this study*. Molecular weights and electric charges have been listed together with each structure. Note that $SOD1^{barrel}$ and the core mutation $SOD1^{I35A}$ have the same structure when folded.

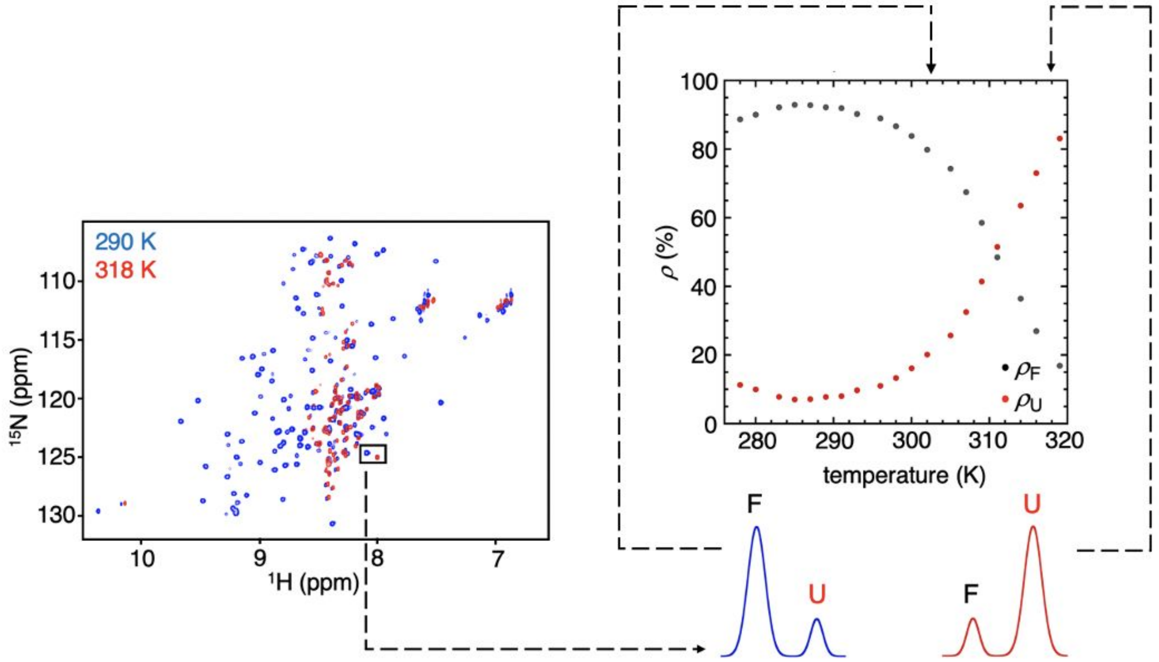

Figure S2. *Quantification of the NMR temperature scan data was done via C-terminal residue Q110***.** The chemical shift of Q110 in SOD1^{I35A} depends on whether the protein is folded (F, blue) or unfolded (U, red). Integration of the respective peak volumes allows for direct quantification of the populations, p_U and p_F, at each temperature according to Eq. 1 in the main text. By plotting the population change as a function of temperature, Eqs. 4 and 5 can be used to determine various thermodynamic parameters of the system, such as the folding equilibrium constant, $K_{U\text{-}F}$, and Gibbs free energy, $\Delta G°_{U\text{-}F}$.

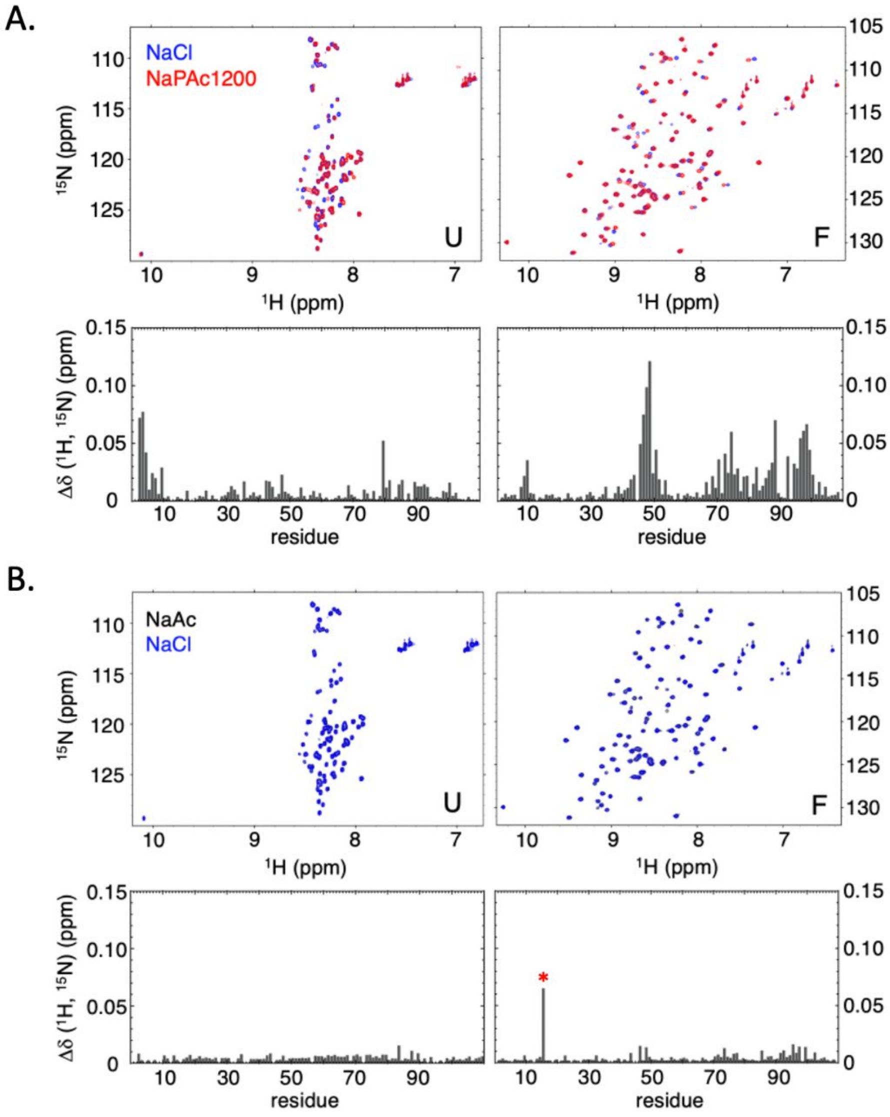

Figure S3. *Comparison of NMR spectra and chemical shift perturbations in different salt environments*. A. Overlapping NMR spectra of SOD1^{barrel} (F) and SOD1^{I35A} (U) in 100 mM NaCl (blue) and 100 mM NaPAc1200 (red). Note that, because line broadening is observed for 100 mM NaPAc1200, the contours have been optimized for peak visibility and therefore differ between the two spectra. The bar plots show the chemical shift differences along the protein sequence. The results are similar to those observed for 100 mM NaAc, as described in the main text. B. Overlapping the spectra of 100 mM NaCl with that of the proteins in 100 mM NaAc (black) shows that there are no major differences between the two monovalent salts. Marked with a red asterisk is G16 that overlaps with other cross-peaks, preventing us from drawing any conclusions based on its chemical shift change.

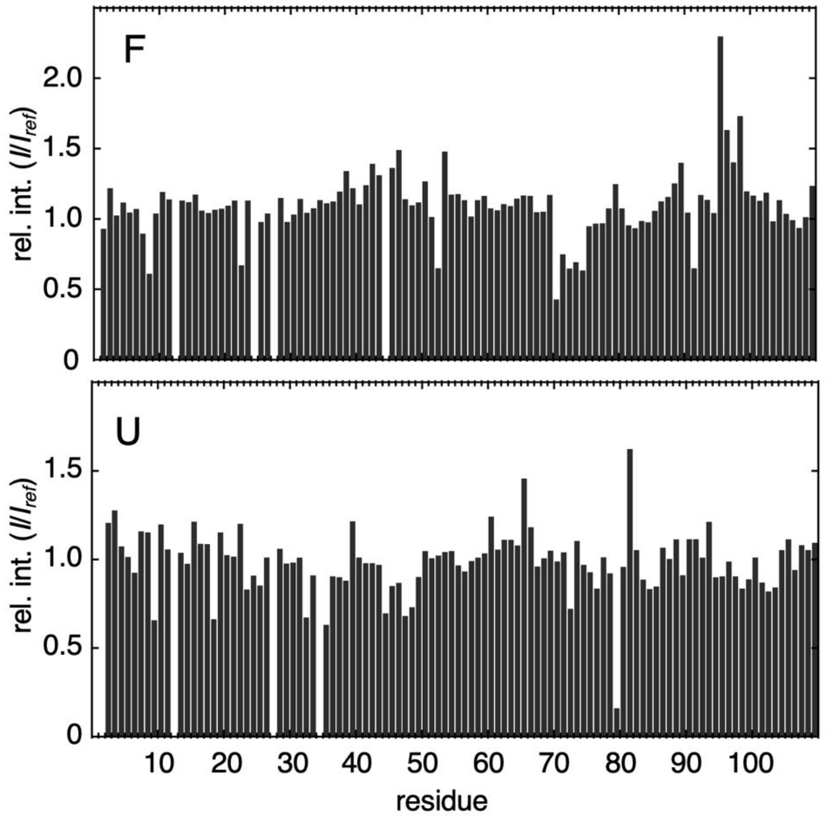

Figure S4. *Addition of NaPAc1200 induces linewidth effects in folded (F) and unfolded (U) SOD1^{barrel}*. The bar plots show the intensity ratios of SOD1^{barrel} in 100 mM NaPAc1200 compared to 100 mM NaCl. Both an increase and decrease in peak intensities can be observed along the sequence, with the largest effect being observed for loops 6 and 7 in the folded protein.

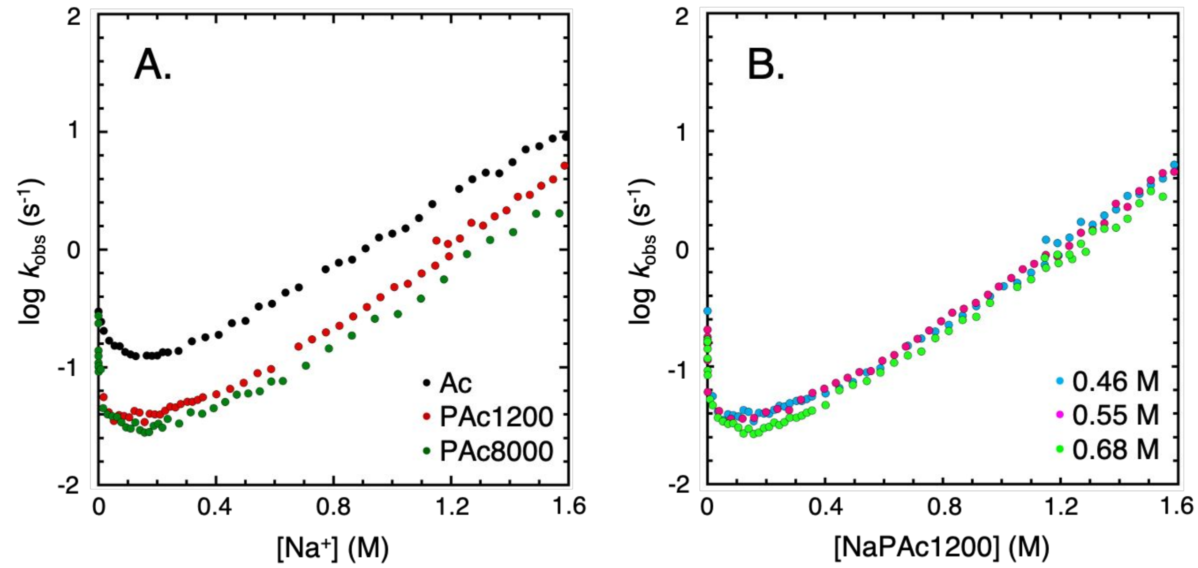

Figure S5. *Control experiments with varying Na^+ concentrations.* A. The plot shows the raw data used in Fig. 4, i.e., before normalization and slope subtraction. The refolding rate of $SOD1^{barrel}$ was studied in 0-1.6 M NaAc (black), NaPAc1200 (red) and NaPAc8000 (green). B. The refolding rate of $SOD1^{barrel}$ was also studied under different urea concentrations, while the NaPAc1200 concentrations were kept constant. No differences can be observed at 0.46 M (light blue) and 0.55 M urea (cerise), except for at 0 M NaPAc1200. However, there is a slight vertical offset at 0.1-0.4 M NaPAc1200 in 0.68 M urea (neon green). This could suggest that at these concentrations the destabilization of urea and NaPAc1200 are complementary, while at higher concentrations the stabilizing screening component of NaPAc1200 would counteract the urea destabilization. However, the observed difference is of the same magnitude as the error of the measurements, making it difficult to draw any conclusions from it. The kinetic data presented in the main text were acquired with 0.46 M urea in the refolding mix.

Supporting Tables

Table S1. Thermodynamic parameters derived from NMR temperature scans, using the model protein $SOD1^{I35A}$, and folding kinetics, using $SOD1^{barrel}$.

Value/co-solute	MES	NaCl	NaAc	NaPAc1200	NaPAc8000	A2780$^{\perp}$
$\Delta G°$ (25°C), kJ/mol	-4.6 ± 0.1$^{¢}$	-2.2 ± 0.1	-3.0 ± 0.1	0.66 ± 0.1	2.5 ± 0.1	-1.2 ± 0.1
$\Delta H°$ (25°C), kJ/mol	-73 + 1	-81 ± 3	-78 ± 5	-77 ± 1	-84 ± 2	-106 ± 8
$\Delta S°$ (25°C), kJ/mol K	-0.23 ± 0.01	-0.26 ± 0.01	-0.25 ± 0.02	-0.26 ± 0.03	-0.29 ±0.01	-0.35 ± 0.03
$\Delta G°$ (37°C), kJ/mol	-0.48 ± 0.1	2.95 ± 0.1	2.38 ± 0.1	5.37 ± 0.1	7.95 ± 0.1	5.34 ± 0.2
$\Delta H°$ (37°C), kJ/mol	-145 ± 6	-182 ± 6	-198 ± 9	-157 ± 6	-182 ± 8	-221 ± 14
$\Delta S°$ (37°C), kJ/mol K	0.46 ± 0.02	-0.60 ± 0.02	-0.64 ± 0.03	-0.52 ± 0.02	-0.61 ± 0.03	-0.73 ± 0.05
$\Delta G°_{max}$, kJ/mol	-5.9 ± 0.1	-3.4 ± 0.1	-4.0 ± 0.1	-0.8 ± 0.1	1.0 ± 0.1	-3.1 ± 0.1
$\Delta H°$ (T_m), kJ/mol	-151 ± 2	-134 ± 4	-157 ± 10	-58 ± 1	-11 ± 4	-134 ± 11
$\Delta S°$ (T_m), kJ/mol K	-0.48 ± 0.01	-0.44 ± 0.01	-0.51 ± 0.03	-0.19 ± 0.01	-0.04 ± 0.01	-0.44 ± 0.04
ΔC_p, kJ/K	-6.0 ± 0.2	-8.4 ± 0.6	-10.0 ± 1.2	-6.7 ± 0.2	-8.2 ± 0.4	-9.6 ± 1.2
T_m, °C	37.8 ± 0.4	31.2 ± 0.4	32.8 ± 0.3	22.0 ± 0.9	16.1$^{\#}$	27.8 ± 0.5
T_c, °C	-9.8 ± 1.0	0.4 ± 0.5	2.3 ± 0.6	5.0 ± 0.8	n.a.	0.5 ± 0.5
T_{max}, °C	13.6 ± 0.5	15.7 ± 0.3	17.5 ± 0.3	13.5 ± 0.5	14.6 ± 0.4	14.1 ± 0.3
$\log k_f^{H2O}$ §	-0.13 ± 0.02	-0.54 ± 0.02	-0.43 ± 0.02	-0.99 ± 0.04	-1.17 ± 0.05	n.a.
m_f, M^{-1} §	-0.84 ± 0.02	-0.82 ± 0.02	-0.87 ± 0.02	-0.84 ± 0.05	-0.77 ± 0.06	n.a.
$\log k_u^{H2O}$ §	-4.08 ± 0.16	-4.08 ± 0.08	-4.04 ± 0.15	-3.98 ± 0.14	-4.10 ± 0.15	n.a.
m_u, M^{-1} §	0.29 ± 0.03	0.28 ± 0.01	0.28 ± 0.03	0.27 ± 0.03	0.28 ± 0.03	n.a.
M_p, M §	3.5 ± 0.2	3.2 ± 0.1	3.1 ± 0.2	2.7 ± 0.2	2.8 ± 0.2	n.a.
$\log K_{U-F}^{H2O}$ §	3.95 ± 0.16	3.54 ± 0.08	3.61 ± 0.15	2.99 ± 0.14	2.92 ± 0.16	n.a.
$\Delta G°_{kin}$(25°C), kJ/mol§	-22.5 ± 0.9	-20.2 ± 0.5	-20.6 ± 0.9	-17.0 ± 0.8	-16.7 ± 0.9	n.a.

§ Kinetic parameters derived from stopped-flow measurements using the model protein $SOD1^{barrel}$ (at 298 K).
¢ Errors are estimated from fitting uncertainties.
$^{\perp}$ Values from Danielsson, et.al, PNAS 2015.
\# T_m is here extrapolated from the approximately linear increase in $\Delta G°$ above 27 °C.

Table S2. Parameters used when docking PAc1200 to SOD1^{barrel} in HADDOCK 2.4.

**Interaction interface**	
Active residues:	K9, G10, G44, H46, V47, H48, G49, A50, G51, I69, D71, V73, I74, S75, L76, S77, D79, H80, I83, L87, V88, V89, A93, A95, G96, A97, G98, S99, R100, L101
Interaction interface cutoff (passive residues):	6.5 Å
**Sampling parameters**	
Number of structures for rigid body docking:	10000
Number of structures for semi-flexible refinement:	400
Number of structures for the final refinement:	400
**Clustering parameters**	
Clustering method:	RMSD
RMSD cutoff for clustering:	2.0
**Scoring parameters**	
Evdw 1:	1.0
Eelec 3:	0.1
**Advanced sampling parameters**	
Initial temperature for second TAD cooling step with flexible side-chain at the interface:	500
Initial temperature for third TAD cooling step with fully flexible interface:	300
Number of MD steps for rigid body high temperature TAD:	0
Number of MD steps during first rigid body cooling stage:	0
